# Comparative Microscopic, Transcriptome and IAA Content Analyses Reveal the Stem Growth Variations in Two Cultivars *Ilex verticillata*

**DOI:** 10.3390/plants12101941

**Published:** 2023-05-10

**Authors:** Sini Qin, Siyi Fu, Ying Yang, Qiumin Sun, Jingqi Wang, Yanling Dong, Xinyi Gu, Tao Wang, Xiaoting Xie, Xiaorong Mo, Hangjin Jiang, Youxiang Yu, Jijun Yan, Jinfang Chu, Bingsong Zheng, Yi He

**Affiliations:** 1 State Key Laboratory of Subtropical Silviculture, Zhejiang A&F University, Hangzhou 311300, China; qinsini98@163.com (S.Q.); fusiyis@163.com (S.F.); sky_yangying@163.com (Y.Y.); sunqiumin99@163.com (Q.S.); jingqiwang1999@163.com (J.W.); 17867963651@163.com (Y.D.); guxinyi200715@163.com (X.G.); wangtao042500@163.com (T.W.); xiexiaoting@stu.zafu.edu.cn (X.X.); bszheng@zafu.edu.cn (B.Z.); 2 Zhejiang Provincial Key Laboratory of Forest Aromatic Plants-Based Healthcare Functions, Zhejiang A&F University, Hangzhou 311300, China; 3 National Forestry and Grassland Administration (NFGA) Research Center for Ilex, Hangzhou 311300, China; 4 State Key Laboratory of Plant Physiology and Biochemistry, College of Life Science, Zhejiang University, Hangzhou 310058, China; xiaorong@zju.edu.cn; 5 Center for Data Science, Zhejiang University, Hangzhou 310058, China; jianghj@zju.edu.cn; 6 National Centre for Plant Gene Research (Beijing), Institute of Genetics and Developmental Biology, Chinese Academy of Sciences, Beijing 100101, China; yanjijun@genetics.ac.cn (J.Y.); jfchu@genetics.ac.cn (J.C.); 7 University of Chinese Academy of Sciences, Beijing 100049, China

**Keywords:** *Ilex verticillata*, stem, xylem, transcriptome, auxin, IAA content

## Abstract

*Ilex verticillata* is not only an excellent ornamental tree species for courtyards, but it is also a popular bonsai tree. ‘Oosterwijk’ and ‘Red sprite’ are two varieties of *Ilex verticillata*. The former has a long stem with few branches, while the latter has a short stem. In order to explain the stem growth differences between the two cultivars ‘Oosterwijk’ and ‘Red sprite’, determination of the microstructure, transcriptome sequence and IAA content was carried out. The results showed that the xylem thickness, vessel area and vessel number of ‘Oosterwijk’ were larger than in ‘Red sprite’. In addition, our analysis revealed that the differentially expressed genes which were enriched in phenylpropanoid biosynthesis; phenylalanine metabolism and phenylalanine, tyrosine and tryptophan biosynthesis in the black and tan modules of the two varieties. We found that *AST*, *HCT* and *bHLH 94* may be key genes in the formation of shoot difference. Moreover, we found that the IAA content and auxin-related DEGs *GH3.6*, *GH3*, *ATRP5*, *IAA27*, *SAUR36-like*, *GH3.6-like* and *AIP 10A5-like* may play important roles in the formation of shoot differences. In summary, these results indicated that stem growth variations of ‘Oosterwijk’ and ‘Red sprite’ were associated with DEGs related to phenylpropanoid biosynthesis, phenylalanine metabolism and phenylalanine, tyrosine and tryptophan biosynthesis, as well as auxin content and DEGs related to the auxin signaling pathway.

## 1. Introduction

The whorled holly (*Ilex verticillata*), also known as north American holly, is an excellent ornamental fruit tree species. ‘Oosterwijk’, the most popular whorled holly variety in the cut branch market, has long branches which provide a good basis for the shape of the cut branches. Another variety, ‘Red sprite’, has a short plant type with many branches and is mainly used in the bonsai market. Plant height is a key agronomic trait closely related to plant structure, photosynthetic efficiency and productivity [1]. Height is determined by several developmental factors such as the number of phytomers and whether a plant exhibits determinate growth or indeterminate growth [2]. Stem elongation plays an important role in determining plant height [3]. The stem consists of nodes and internodes that form the central axis of the plant’s stem system. In addition to supporting the plant, the vascular system of the stem also functions to transport water and mineral nutrients [4]. The elongation of plant stems is caused by a combination of cell division and cell elongation, and elongation growth usually occurs during the transition from primary to secondary stem growth [5]. Plant stem elongation is regulated and controlled by various internal and external factors, among which the internal regulation of endogenous plant hormones such as gibberellin (GAs), brassinosteroid (BRs), strigolactone (SL) and auxin play an important role.

Auxin plays an important role in regulating stem growth and development. Auxin promotes cell division and elongation by regulating the cell cycle, promoting cell wall extension and inducing RNA and protein synthesis. The experiments of suspension-cultured Acer pseudoplatanus cells and tobacco cell lines showed auxin-regulated plant cell division [6,7]. Different types of auxins have different receptors and different signaling pathways leading to different controls of cell division. The study showed that *ABP1* is the receptor for NAA and promotes cell elongation, while Rx is the receptor for 2,4-D and induces the cell division process. Different Arabidopsis Aux/IAA mutants, such as *axr2/iaa7*, *axr5/iaa1*, *axr3/iaa17* and *shy2/iaa3*, all have cell expansion defects, indicating that auxin induces cell expansion by degrading Aux/IAAs [8,9,10]. The cell wall limits the expansion of cells and auxin promotes cell expansion by promoting cell wall stretching and increasing cell wall plasticity [11]. In addition, auxin could induce changes in cell wall components, activate the expression of cell wall-related genes, stimulate the synthesis of proton pumps and cause apoplast acidification, thus changing the structure of the cell wall. The expression and activity of EXP (cell wall modification protein) are affected by auxin. *IAA7-* and *bnaarf6/8-*dependent auxin signals control stem elongation and plant height by regulating the transcription of the *BnaEXPA5* gene [12]. Auxin induces the synthesis of RNA and proteins, providing raw materials for the synthesis of protoplasts and cell walls, thus promoting growth [13]. During the treatment of maize coleoptile, the researchers found that the synthesis of growth-limiting proteins induced by auxin could be inhibited by imine cyclohexanone and indeed plant growth was affected accordingly [14]. In addition, genes related to the auxin signal transduction pathway (such as *TIR1*, *ARF*, *GH3* and *SAUR*) have also been reported to be involved in plant stem growth. A study showed that *PtrFBL1* and *PtrFBL7* of the poplar *TIR1* gene family are expressed in vascular tissue and the cambium of poplar, respectively [15]. In *Cannabis sativa*, *ARF3*, *ARF5*, *ARF7*, *ARF8, ARF10* and *ARF15* may be involved in the formation of roots and stems [16]. In *Arabidopsis thaliana*, *arf6/arf8* double mutants and *arf6* and *arf8* single mutants have twisted leaves and short inflorescence stems [17,18]. In *Eucalyptus grandis*, *ARF10* and *ARF19A* may participate in the differentiation of meristem cambium cells into xylem cells [19]. In *Salvia miltiorrhiza*, *ARF16* participates in the development of the stem [20]. In *Brachypodium distachyon*, *ARFs* are also expressed in the short stem [21]. Low expression of *SAUR* and high expression of *GH3* may be related to the reduction of plant height of cotton dwarf mutants [22]. Auxin regulates the growth of plant stems by acting synergistically with other plant hormones [23]. Auxin can reduce the inactivation of active gibberellin by inducing the conversion of GA_20_ into active gibberellin GA_1_, inhibiting the conversion of GA_1_ into GA_8_ (no physiological activity), maintaining a higher level of GA_1_ in the stem and promoting stem elongation. Furthermore, stem elongation can also be promoted by the synergistic effect between auxin and brassinosteroid (BR). Polar transport of auxin can be regulated by BR, which results in the uneven distribution of auxin in crop tissues, thereby regulating crop stem development [24]. All the above results suggested that auxin-mediated pathways will affect stem formation to varying degrees and play important roles in the maintenance and differentiation of stem cells [25,26,27].

In recent years, microstructures combined with transcriptome sequencing to observe stem development differences and screen key genes have been widely used. In two alfalfa genotypes, microstructural observations revealed different lignin contents between the two varieties and the stem lignin synthesis gene was screened by transcriptome data [28]. In *Cynodon dactylon* L., microstructure and transcriptome sequencing were used to study the genetic control of the tiller angle and stem growth [29]. The differences in stem bending between Xixia Yingxue (curved) and Hongfeng (erect) of two *Paeonia lactiflora Pall.* varieties were studied [30]. It was found that there were differences in the degree of secondary cell wall thickening and the number of thickened secondary cell wall layers and 10 key genes related to lignin synthesis were screened by transcriptome sequencing. However, the underlying molecular mechanism of stem variations between *Ilex verticillata* ‘Oosterwijk’ and ‘Red sprite’ is still unclear. In order to explore the mechanism of plant height and stem growth difference between the two varieties, we carried out phenotypic analysis, transcriptome sequencing and endogenous IAA content determination of ‘Oosterwijk’ and ‘Red sprite’ in three growth stages. We found that the phenotypes of the two cultivars were quite different. Through comparative transcriptome analysis combined with the results of endogenous IAA content determination, the difference in auxin content and the expression changes of related genes may be the key factors leading to the difference in stem growth between the two cultivars. This can provide a theoretical basis for plant type regulation and the variety improvement of whorled holly and a foundation for the next step of cultivation of whorled holly.

## 2. Results

### 2.1. Morphological and Histological Analysis of the Stem in ‘Oosterwijk’ and ‘Red Sprite’

**Figure 1:**
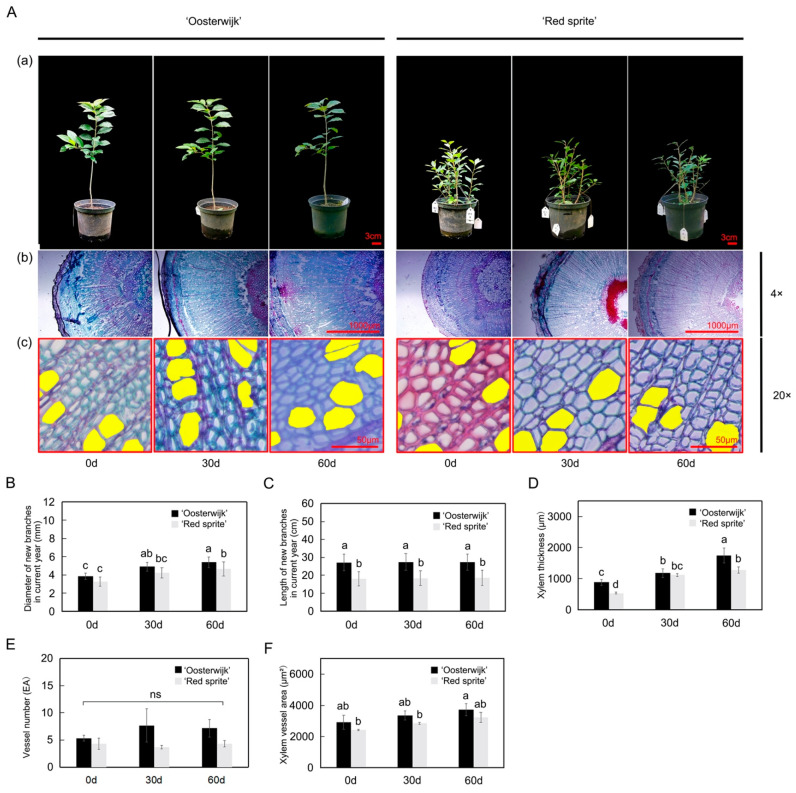
Phenotypic analyses and xylem changes of new branches in the current year of *Ilex verticillata* ‘Oosterwijk’ and ‘Red sprite’. (**A**) Observation of the phenotypes. (**a**) The seedlings of ‘Oosterwijk’ and ‘Red sprite’. Scale bar = 3 cm and (**b**) observation of the xylem thickness of the two cultivars demonstrates the distance from pith to phloem and the xylem thickness. The sections were observed under a positive fluorescence microscope at ×4 magnification. Scale bar = 1000 μm; (**c**) observation of xylem vessels of the two cultivars, the yellow area indicates the vessel area. The sections were observed under a positive fluorescence microscope at 20 magnification. Scale bar = 50 μm. (**B**) Diameter changes of new branches in the current year in two *Ilex verticillata* cultivars. (**C**) Length changes of new branches in the current year in two *Ilex verticillata* cultivars. (**D**) Xylem thickness in two *Ilex verticillata* cultivars. The calculation was performed by Image J software based on every three slices. (**E**) Xylem vessel numbers in two *Ilex verticillata* cultivars. (**F**) Xylem vessel area in two *Ilex verticillata* cultivars. The vessel area was measured by Image J software. Bars represent the mean ± SD of at least three independent experiments. Different letters indicate significantly different values (*p* < 0.05) by Tukey’s test.

To compare the differences in stem growth between ‘Oosterwijk’ and ‘Red sprite’, we analyzed the stem phenotypes in the different growth phases (every 30 days as a harvesting time point) from 29 May 2021 to 31 July 2021 (Figure 1A). The results showed that there are significant differences in the way of branching and the length of stem between them. Compared with 0 days, the stem diameter of new branches of ‘Oosterwijk’ increased by 27.5% and 39.7%, respectively, and that of ‘Red sprite’ increased by 30.3% and 42.7% in 30 and 60 days (Figure 1B), respectively. In addition, there was no significant difference in stem diameter between the two varieties in the early stage, but in the third period, the stem diameter of ‘Oosterwijk’ was significantly larger than that of the ‘Red sprite’. We also discovered that the length of the new branch of ‘Oosterwijk’ was longer than that of ‘Red sprite’ (about 9 cm), but there was no significant difference in the lengths of the new shoots of the two varieties in 0 d vs. 30 d vs. 60 d (Figure 1C). From subsequent observations, both ‘Oosterwijk’ and ‘Red sprite’ appeared to have new shoots in autumn (Supplementary Figure S1). The autumn new shoot of ‘Oosterwijk’ was greener, thinner and longer than ‘Red sprite’.

To further explore the structural differences between the new stems of the two varieties, we observed the cross-section of the new stems through paraffin sections (Figure 1A). The results showed that compared with the xylem of the ‘Red sprite’ stem, the xylem of ‘Oosterwijk’ was thicker and grew faster. At 60 days, the thickness of the xylem of ‘Oosterwijk’ was 1743 μm, while the thickness of the xylem of ‘Red sprite’ was 1284 μm (Figure 1D). We also found that the xylem area of the ‘Oosterwijk’ stem contains more ducts: about 1.0 times that of the ‘Red sprite’ (Figure 1E). The xylem vessel area of ‘Oosterwijk’ was always higher than that of ‘Red sprite’ (about 500 μm^2^) and the area of xylem vessel in ‘Oosterwijk’ increased significantly at 60 days, but there was no significant difference in ‘Red sprite’ (Figure 1F). In addition, the longitudinal section of the stem was observed and it was found that the wood layer of ‘Red sprite’ was more compact and detailed (Supplementary Figure S2).

### 2.2. Analysis of Differentially Expressed Genes

In order to further explore the mechanism of the stem growth difference between the ‘Oosterwijk’ and ‘Red sprite’, 18 gene expression profiles were constructed from the two genotypes with three replicates, and with each at three growth stages (0, 30 and 60 DAG). Each cDNA library produced 31.67–47.36 million Valid Reads and the proportion of Valid Reads reached more than 91%, including 115.38 Gb with an average of 6.41 Gb/library and a total of 30267 transcripts were obtained. The clean reads were compared to the genome of *Ilex verticillata* for comparative analysis and it was found that the comparison rate between the samples and the reference genome reached more than 88%, that the average comparison rate was 92.68% and that most of the comparison rates reached more than 90% (Table S1).

**Figure 2:**
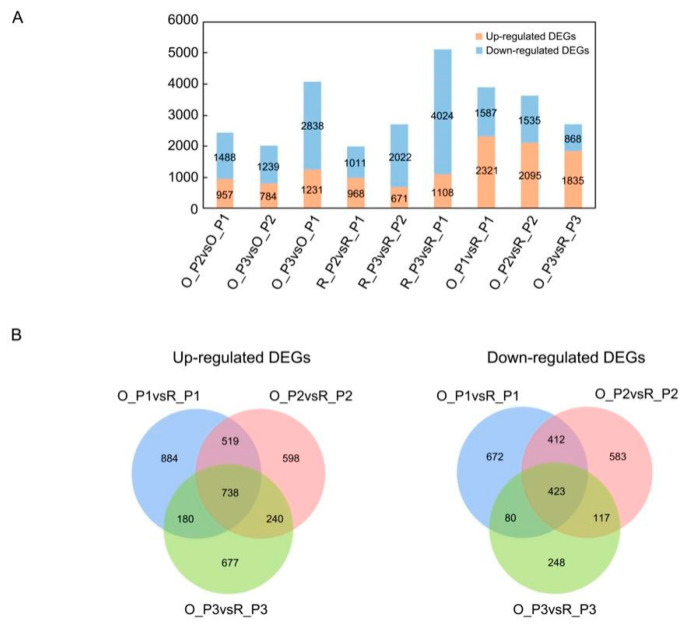
The identification of differentially expressed genes (DEGs) and co-expressed gene modules from WGCNA analysis. (**A**) Up- and down-regulation DEGs in each comparison group. O_P2 vs. O_P1, O_P3 vs. O_P2, O_P3 vs. O_P1, R_P2 vs. R_P1, R_P3 vs. R_P2, R_P3 vs. R_P1, O_P1 vs. R_P1, O_P2 vs. R_P2 and O_P3 vs. R_P3; O indicates ‘Oosterwijk’ and R indicates ‘Red sprite’; P1, P2 and P3 indicate Phase1 (0 d), Phase2 (30 d) and Phase3 (60 d). The blue bars represent the number of down-regulated genes and the orange bars represent the number of up-regulated genes. (**B**) Venn plots of up- and down-regulated DEGs in O_P1 vs. R_P1, O_P2 vs. R_P2 and O_P3 vs. R_P3 groups.

**Figure 3:**
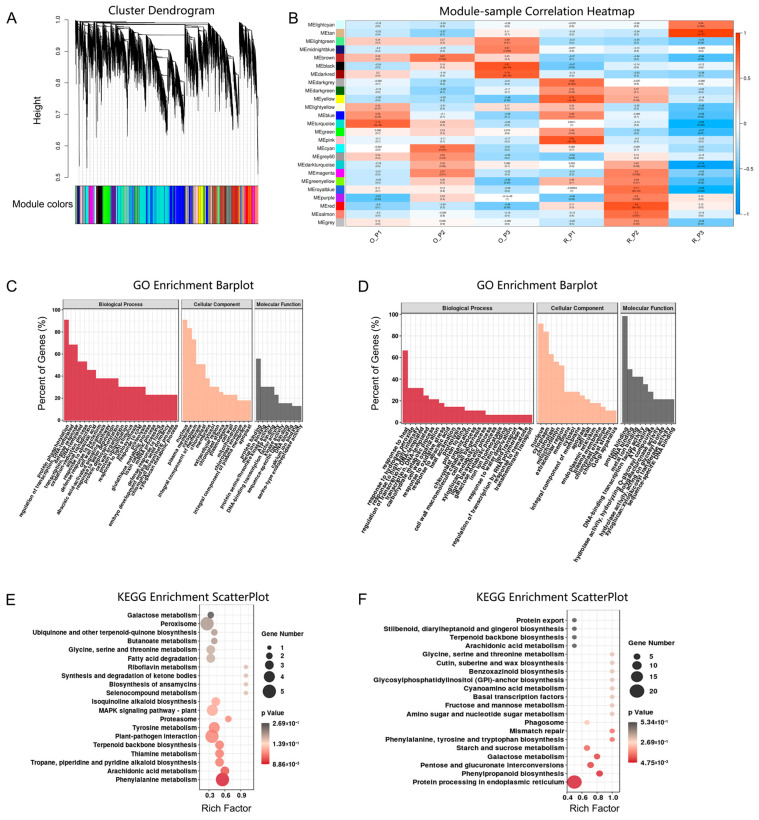
Co−expression module analysis of the transcriptome in stems tissue. (**A**) Co−expressed gene modules detection by gene cluster dendrograms. (**B**) Co−expressed gene module−sample associations revealed by the Person correlation coefficient. The leftmost color column indicates different co−expression modules. The number in the figure indicates the correlation between the modules and samples and the numbers in the parentheses are the correlation *p* values. (**C**) GO functional enrichment analysis of DEGs in the black module in the O_P3 vs. R_P3 group. (**D**) GO functional enrichment analysis of DEGs in the tan module in the O_P3 vs. R_P3 group. (**E**) KEGG enrichment analysis of DEGs in the black module. (**F**) KEGG functional enrichment analysis of DEGs in the tan module. O indicates ‘Oosterwijk’ and R indicates ‘Red sprite’; P3 indicates Phase3 (60 d).

To understand the changes in gene expression during the growth transition between two cultivars, we identified differentially expressed genes (DEGs) between one time point and the previous time point. According to q-adjust < 0.05, the difference multiple of up-down is two, namely |log2 fc| ≥ 1, and the up- and down-regulation of differentially expression genes were obtained (Figure 2A and Supplementary Figure S3). In the stem development phase of ‘Oosterwijk’ and ‘Red sprite’, down-regulated genes were more than up-regulated genes. In O_P3 vs. O_P1 and R_P3 vs. R_P1, the number of up-regulated and down-regulated genes had the largest difference, with 1231 and 1108 up-regulated genes, respectively. At the same time, there were 4024 down-regulated genes in ‘Red sprite’, but only 2838 down-regulated genes in ‘Oosterwijk’. However, the number of up-regulated DEGs was more than the number of down-regulated DEGs in O_P1 vs. R_P1, O_P2 vs. R_P2 and O_P3 vs. R_P3. In O_P1 vs. R_P1, 2321 genes were up-regulated while the number of upregulated genes decreased to 1835 in O_P3 vs. R_P3. This appears to be due to fewer downregulated genes at 60 days in ‘Red sprite’. The results of the Venn Diagrams illustrated the overlaps and differences between the different comparison groups (Figure 3B). In O_P1 vs. R_P1, O_P2 vs. R_P2 and O_P3 vs. R_P3, there were 738 common upregulated DEGs and 884, 598 and 677 up-regulated specific DEGs. In addition, there were 423 common down-regulated DEGs and 672, 583, 248 specific down-regulated DEGs (Figure 2B).

### 2.3. GO Classification and KEGG Analysis of Differentially Expressed Genes

To explore the major functional categories and enrichment pathways of DEGs, the GO classification and KEGG analysis of DEGs was performed. The DEGs were annotated in the database. Based on GO analysis, we found that DEGs were annotated to the biological (physiological) process (GO:0008150), regulation of transcription, DNA-templated (GO:0006355) and transcription, DNA-templated (GO:0006351) under biological process (BP), nucleus (GO:0005634), plasma membrane (GO:0005886) and cytoplasm (GO:0005737) under cellular component (CC) and molecular function (GO:0003674) and protein binding (GO:0005515) under molecular function (MF) (Supplementary Figures S4–S6). There were 280 genes related to auxin or tryptophan among these annotations under BP, CC and MF.

According to the KEGG annotation results, these unigenes were classified and graded (Supplementary Figures S7–S9). In the O_P2 vs. O_P1, O_P3 vs. O_P2, O_P3 vs. O_P1, R_P2 vs. R_P1, R_P3 vs. R_P2 and R_P3 vs. R_P1 groups, DEGs were mainly enriched in plant hormone signal transduction (map04075), starch and sucrose metabolism (map00500), MAPK signaling pathway (map04016) and protein processing in the endoplasmic reticulum (map04141). Among them, O_P2 vs. O_P1 and R_P2 vs. R_P1 were mostly enriched in diterpenoid biosynthesis (map00904). In the O_P1 vs. R_P1, O_P2 vs. R_P2 and O_P3 vs. R_P3 groups, DEGs were mainly enriched in phenylpropanoid biosynthesis (map00940), flavonoid biosynthesis (map00941), zeatin biosynthesis (map00908), galactose metabolism (map00052) and phenylalanine metabolism (map00360). In addition, we discovered that the pathways related to tryptophan (map00380, map00400) were shown in the O_P2 vs. R_P2 and O_P3 vs. R_P3 groups. (Supplementary Figures S7–S9). These results indicated that the differential regulatory mechanism is relevant in phenylpropanoid biosynthesis, phenylalanine metabolism related to lignin synthesis and the pathway related to auxin, which might be the cause of differences in stem growth.

### 2.4. Co-Expression Network Analysis of Genes in the Module

To understand the association between stem difference and phenylpropanoid, phenylalanine more comprehensively and auxin gene regulation, a weighted gene co-expression network analysis (WGCNA) was performed. A total of 24,196 genes were grouped into 25 modules (Figure 3A). The turquoise module contained the most genes (5959 genes) and the grey module contained the least genes (24 genes) (Table S2). In addition, to find the key modules related to the stem differences of ‘Oosterwijk’ and ‘Red sprite’, the correlation between modules and xylem development was analyzed. There were significant differences in xylem development in the third stage, so we chose the modules with the strongest correlation at 60 d. The result indicated that two modules (the. black module and the tan module) were the critical modules and will be identified for further analysis (Figure 3B).

In the black module, based on GO analysis the majority of DEGs were annotated to protein phosphorylation, defense response and regulation of transcription, DNA-templated under biological process (BP), nucleus, plasma membrane and cytoplasm under cellular component (CC) and protein binding and ATP binding under molecular function (MF) (Figure 3C). In addition, DEGs were mainly enriched in phenylalanine, tyrosine and tryptophan biosynthesis (Figure 3E). In the tan module, based on GO analysis, the majority of DEGs were annotated in response to heat, in response to high light intensity and in the regulation of transcription. Furthermore, they were DNA-templated under biological process (BP); the nucleus, cytoplasm and chloroplast were under cellular component (CC) and protein binding and DNA binding were under molecular function (MF) (Figure 3D). Moreover, DEGs were mainly enriched in protein processing in the endoplasmic reticulum, phenylpropanoid biosynthesis and phenylalanine, tyrosine and tryptophan biosynthesis (Figure 3F). Therefore, the results of WGCNA suggested that phenylpropanoid biosynthesis and phenylalanine, tyrosine and tryptophan biosynthesis might be the important reason for stem growth differences.

### 2.5. Construction of the Correlation Network of DEGs in the Module

**Figure 4:**
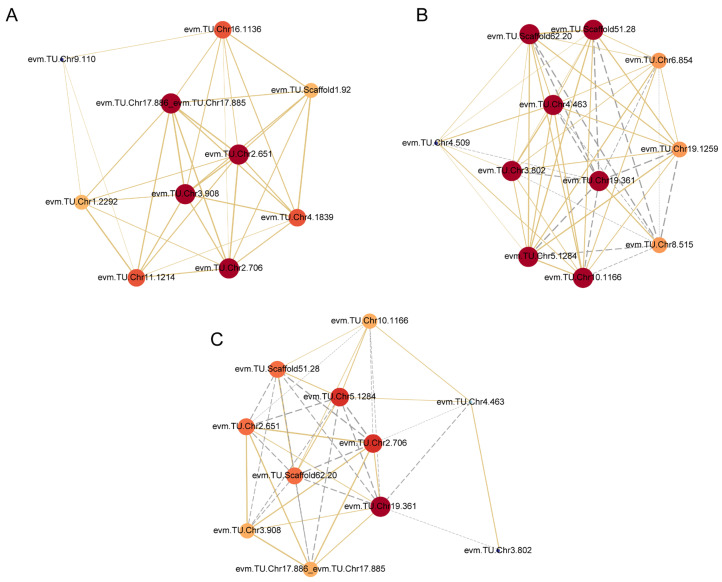
Correlation network analysis based on the DEGs in two modules in the O_P3 vs. R_P3 group. (**A**) Correlation network of screened DEGs in the black module. (**B**) Correlation network of screened DEGs in the tan module. (**C**) Correlation network of screened hub DEGs in two modules. O indicates ‘Oosterwijk’ and R indicates ‘Red sprite’; P3 indicates Phase3 (60 d). The size and color of the circle represent the centrality of the gene. The centrality from blue to red increases gradually. The solid yellow lines reveal positive relations and the grey dotted lines reveal negative relations.

In order to understand the potential regulation mechanism of hub DEGs in two modules, the gene co-expression regulatory networks were constructed according to the data of DEGs from four pathways of phenylpropanoid biosynthesis, phenylalanine, tyrosine and tryptophan biosynthesis, phenylalanine metabolism and tryptophan metabolism. These two networks comprised 21 DEGs and intricate relationships existed among these genes, as shown in Figure 4A,B.

**Figure 5:**
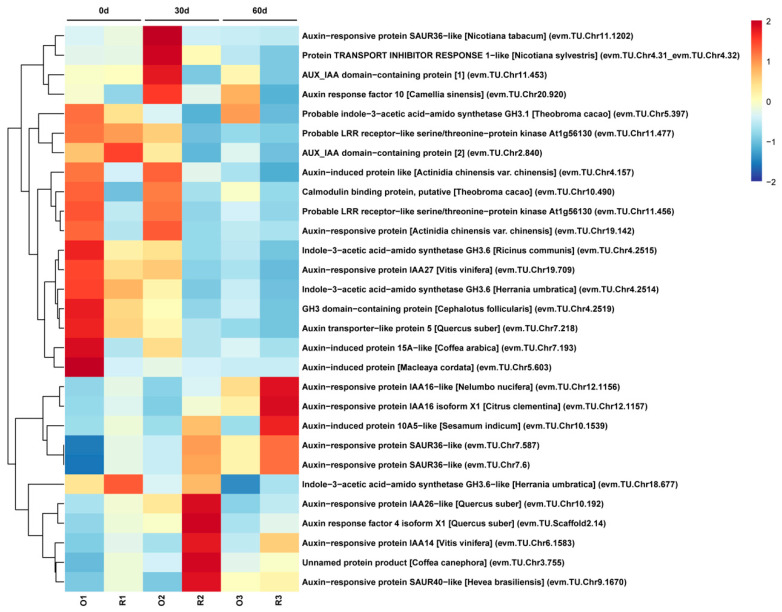
Heatmap of DEGs related to the auxin signaling pathway in the O_P1 vs. R_P1, O_P2 vs. R_P2 and O_P3 vs. R_P3 groups. O indicates ‘Oosterwijk’ and R indicates ‘Red sprite’; P1, P2 and P3 indicate Phase1 (0 d), Phase2 (30 d) and Phase3 (60 d). A scale indicating the color assigned to is shown to the right of the heatmap.

In the black module, four potential genes including peroxidase 25 (evm.TU.Chr3.908), putative caffeoyl-CoA O-methyltransferase At1g67980 (evm.TU.Chr17.886, evm.TU.Chr17.885), aspartate aminotransferase, cytoplasmic (evm.TU.Chr2.706) and transcription repressor MYB6-like (evm.TU.Chr2.651) were central in the network (Figure 5A). We also discovered that the expression levels of these central genes were all higher in ‘Oosterwijk’ compared with ‘Red sprite’ in the O_P3 vs. R_P3 group. In the tan module, seven potential genes including shikimate O-hydroxycinnamoyltransferase (evm.TU.Chr19.361), cytochrome b-c1 complex subunit 8 (evm.TU.Chr4.463), aldehyde dehydrogenase family 2 member C4-like isoform X1 (evm.TU.Chr3.802), sec-independent protein translocase protein like (evm.TU.Chr10.1166), transcription factor bHLH94 (evm.TU.Chr5.1284) and transcription factor bHLH96 (evm.TU.Scaffold62.20, evm.TU.Scaffold51.28) were central to the network (Figure 4B). There were both positive and negative regulatory relations between them. For example, there was a positive regulatory relationship between evm.TU.Chr4.463 and evm.TU.Scaffold62.20 but there was a negative relationship between evm.TU.Chr4.463 and evm.TU.Scaffold51.28. In addition, the expression levels of these genes were all lower in ‘Oosterwijk’ compared with ‘Red sprite’ except for gene evm.TU.Chr19.361 in the O_P3 vs. R_P3 group. Therefore, these genes may be the potential core genes for stem growth.

Accordingly, four hub-screened genes in the black module and seven hub-screened genes in the tan module were selected to construct a general network (Figure 4C). Correspondingly, three genes including evm.TU.Chr19.361 (*HCT*), evm.TU.Chr2.706 (*AST*) and evm.TU.Chr5.1284 (*bHLH94*) were predicted to be the vital genes of stem growth difference. The *bHLH94* belongs to the *bHLH* transcription factor family, which is the second largest family of transcription factors in plants and participates in multiple developmental processes of plants. Moreover, *HCT* is a key enzyme involved in the lignin biosynthesis pathway which affects the biosynthesis of G/S monolignol. Studies have shown that *AST* is involved in the biosynthesis of phenylalanine, tyrosine and tryptophan and that tryptophan is an important precursor of auxin biosynthesis in plants. There was a positive regulatory relationship between *HCT* and *AST* but there was a negative regulatory relationship between *HCT* and *bHLH94.* In addition, there was a negative regulatory relationship between *AST* and *bHLH94.* In the O_P3 vs. R_P3 group, the expression of *AST* was higher in ‘Oosterwijk’ than that in ‘Red sprite’, but the expression of *HCT* and *bHLH94* were lower in ‘Oosterwijk’ than in ‘Red sprite’. Taken together, these results suggest that *bHLH* transcription factors, lignin synthesis and tryptophan biosynthesis pathways are key factors in stem growth differences between the two cultivars. Among them, *AST* may be a key regulatory gene which may affect the synthesis of tryptophan, resulting in the change of auxin content. It is then suggested that the difference in stem growth between the two varieties may also be related to the auxin content.

### 2.6. Analysis of DEGs Related to the Auxin Signaling Pathway and Endogenous Auxin Content Determination

**Figure 6:**
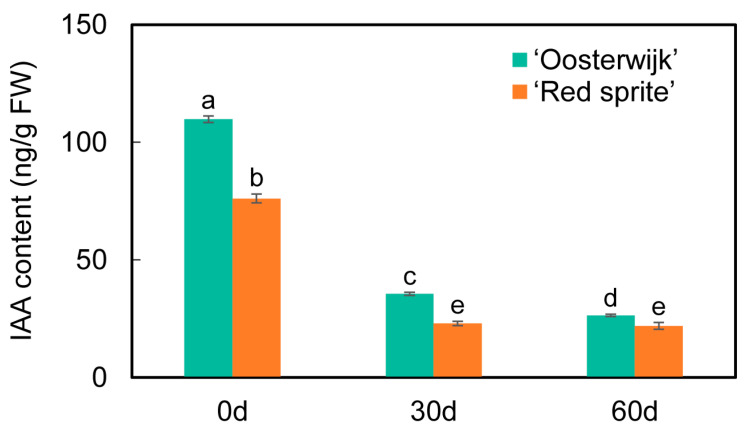
IAA content in the stem of ‘Oosterwijk’ and ‘Red sprite’. Bars represent the mean ± SD of at least three independent experiments. Different letters indicate significantly different values (*p* < 0.05) by Tukey’s test.

To explore the relationship between stem growth and the expression of auxin-related genes, the expression of the genes involved in the auxin signaling pathway was analyzed. The 29 DEGs related to the auxin signaling pathway were screened and selected for further analysis (Figure 5). Among these DEGs in 3 phases, the expression levels of 14 DEGs were higher in ‘Oosterwijk’ at each phase than that compared with ‘Red sprite’ and 9 genes were more highly expressed in ‘Red sprite’. These differential genes are mainly concentrated in auxin synthesis, transport and signal transduction pathways. The *SAUR* genes are the early auxin response gene, which has the fastest and strongest response to auxin and regulates the synthesis and transport of auxin. Our study showed that the expression levels of the three *SAUR* genes (evm.TU.Chr7.587, evm.TU.Chr7.6 and evm.TU.Chr10.1539) in ‘Red sprite’ were higher than those in ‘Oosterwijk’. However, the expression levels of *GH3* genes (evm.TU.Chr4.2515, evm.TU.Chr4.2519 and evm.TU.Chr4.2514), *IAA27* (evm.TU.Chr19.709) and *AUX1* (evm.TU.Chr7.218) were higher in Oosterwijk than that in ‘Red sprite’. These results suggested that the differential expression of DEGs related to the auxin signaling pathway may affect the variation of endogenous auxin content, which in turn leads to the difference in stem growth between the two cultivars. To further understand the relationship between auxin and stem development, the IAA contents of the stem in both cultivars were determined. The endogenous auxin content was always higher in ‘Oosterwijk’ than that in ‘Red sprite’ at each phase (Figure 6). Thus, these results suggested that the auxin content is positively related to the stem growth in the two cultivars.

### 2.7. Analysis of Auxin Content Determination

To further understand the relationship between auxin and stem development, the IAA contents of the stem in both cultivars were determined. The endogenous auxin content was always higher in ‘Oosterwijk’ than that in ‘Red sprite’ at each phase (Figure 6). Thus, these results suggested that the auxin content is positively related to the stem growth in the two cultivars. Based on the determination of IAA content and transcriptome analysis, the nine DEGs including two *GH3.6* genes, *GH3*, *ATRP5/AUX1*, *IAA27*, two *SAUR36-like* genes, *GH3.6-like* and *AIP 10A5-like* were screened and summarized in the auxin signaling pathway (Figure 5). In ‘Oosterwijk’, *GH3*, *IAA27* and *GH3.6-like* were all down-regulated at each phase, while two *SAUR36-like* genes and *AIP 10A5-like* were all up-regulated. In ‘Red sprite’, the up-regulation and down-regulation were basically the same as that of ‘Oosterwijk’. In addition, the expression levels of the influx vector *ATRP5/AUX1* were higher in ‘Oosterwijk’ than that in ‘Red sprite’. Auxin downstream reaction factors of two *GH3.6* genes, *GH3* and *IAA27,* were higher in ‘Oosterwijk’ than in ‘Red sprite’, while the expression levels of *GH3.6-like*, two *SAUR36-like* genes and *AIP 10A5-like* were lower in ‘Oosterwijk’ than that in ‘Red sprite’. Therefore, these results suggested that there were positive correlations between five DEGs (two *GH3.6* genes, *GH3, ATPR5/AUX1* and *IAA27*) and stem growth, while there were negative correlations between four DEGs (*GH3.6-like*, two *SAUR36-like* genes and *AIP 10A5-like*) and stem growth.

## 3. Discussion

### 3.1. Water Transport Capacity of the ‘Oosterwijk’ Stem May Be Stronger Than ‘Red Sprite’

Stem length is one of the main factors that affect plant appearance. Further study on the difference of stem growth will be helpful to improve and produce more suitable products for market. In our study, these results indicated that the pith, xylem thickness and vessel area of ‘Oosterwijk’ were larger than those of ‘Red sprite’. Therefore, the mechanism of the stem growth difference finally leads to the phenotypic differences in two cultivars.

A study suggested that the tracheid bridge (a possible pathway between vessels and tracheid to transport water) may be the dominant pathway for water transport between vessels in some species [31]; indeed xylem vessels played an important role in transporting water. In *Vitis vinifera* L., large vessels were more efficient in the volume of transported water compared to narrower ones [32]. In *Rhizophora apiculata*, increasing the diameter of the vessels can improve water transport efficiency [33]. In *Phyllostachys*, the average vessel diameter of *Phyllostachys bambusoides Sieb*, *Phyllostachys glauca f*, and *Phyllostachys glauca McClure* showed a proportional relationship to the water transport efficiency [34]. In our study, the vessel area of ‘Oosterwijk’ was larger than that of ‘Red sprite’. This result suggested that the water transport capacity of ‘Oosterwijk’ may be stronger than that of ‘Red sprite’. In addition, a study showed that late-successional traits *V.dulitensis* had significantly smaller vessel areas and diameters but higher vessel density [35]. These traits were similar to those of ‘Red sprite’ in this study. Therefore, these results suggested that ‘Red sprite’ may be the later successional species and adopted a conservative resource acquisition and growth strategy.

### 3.2. AST, HCT and bHLH94 May Be the Key Genes to the Regulation of Stem Growth

In the O_P3 vs. R_P3 group, the DEGs related to phenylpropanoid biosynthesis, phenylalanine metabolism, the phenylalanine, tyrosine and tryptophan biosynthesis pathway and transcription factors were selected from the black and tan module. In the black module and the tan module, 10 DEGs and 11 DEGs were screened, respectively. Finally, we obtained three hub genes, namely *AST*, *HCT* and *bHLH94*, that might be related to the regulation of stem growth.

In two contrasting maize inbreds viz., I110 (susceptible) and I172 (tolerant), I110 had a higher *AST* expression level than that of I172, which consumed more energy and oxygen [36]. In *Ilex verticillata*, ‘Oosterwijk’ had the higher *AST* expression level compared with ‘Red sprite’. Thus, this result also suggested that ‘Oosterwijk’, with the high *AST* expression level, may consume more energy and oxygen. In addition, the study in *Dianthus chinensis* suggested that the high expression level of *HCT* may cause high lignin content and huge stems [37]. This finding was consistent with the phenotype of ‘Oosterwijk’ and its high expression level of *HCT*. This suggested that *HCT* may be positively related to stem growth. Moreover, the study showed that the *bHLH* transcription factor played a role in poplar and tomato stem development [38,39]. In *Ilex verticillata*, the expression level of *bHLH94* in ‘Oosterwijk’ was lower than that in ‘Red sprite’, which suggested that *bHLH94* may negatively regulate the development of the xylem and stem. These results suggested that *AST, HCT* and *bHLH94*, as the center of the network, played an important role in the regulation of stem growth and could be used as candidate genes for the further study of *Ilex verticillata* stem growth regulation.

### 3.3. Auxin Affected the Growth and Development of the Stem

The study showed that the change in IAA content may be related to stem growth and the auxin concentration directly affected the development of the stem in soybeans [40]. Another study showed that endogenous auxin levels of a novel spontaneous cucumber mutant (named si107) with short internodes were decreased [41]. In *Ilex verticillata*, the IAA content of ‘Oosterwijk’ was higher than that compared with ‘Red sprite’ in three phases. The stem length of ‘Oosterwijk’ were larger than that of ‘Red sprite’. Thus, these results suggested that the content of IAA was positively related to the growth and development of the stem.

A previous study showed that the treatment of white clover with exogenous IAA will result in the down-regulation of the auxin-responsive genes *GH3.6* and *IAA27* [42]. However, *Arabidopsis thaliana* treated with NAA induced the expression of *GH3.6* [43]. In rice, the study showed that *GH3.6* was highly correlated with mesocotyl elongation [44]. In our study, all IAA pathway-related genes were screened for deeply digging out the relationship between the content of IAA and gene expression levels. The result showed that the *GH3.6* expression level of ‘Oosterwijk’ was higher than that of ‘Red sprite’. In addition, the study showed that the *IAA27* transcript in blueberries was highly accumulated in shoots, but the overexpression of *VcIAA27* in *Arabidopsis* will lead to auxin deficiency and form dwarf plants [45]. Additionally, the study showed that *IAA27* in Arabidopsis was a negative regulator of root branching [46]. In *Ilex verticillata*, the *IAA27* content level of ‘Oosterwijk’ was higher than that of ‘Red sprite’. Moreover, some studies demonstrated that *SAUR36/RAG1* was related to the regulation of hypocotyl elongation in *Arabidopsis thaliana* [47]. In our study, the *SAUR36* expression level of ‘Oosterwijk’ was lower than that of ‘Red sprite’. Furthermore, in *Ilex verticillata*, the expression level of *ATRP5* was higher in ‘Oosterwijk’ than that in ‘Red sprite’ but the expression level of *AIP 10A5-like* was lower in ‘Oosterwijk’ than that in ‘Red sprite’. These results suggested that *GH3.6*, *IAA27* and *ATRP5* may be positively related to the growth and development of the stem, while *SAUR36* and *AIP 10A5-like* may be negatively related to the growth and development of the stem.

## 4. Materials and Methods

### 4.1. Plant Growth Conditions

*Ilex verticillata* ‘Oosterwijk’ and ‘Red sprite’ were cultured at the East-Lake campus of Zhejiang Agricultural and Forestry University in early May 2021. The plant tissues of ‘Oosterwijk’ and ‘Red sprite’ were collected for experiments every month from 29 May 2021 to 31 July 2021. Stems close to the bottom of new branches were collected. The stems for the section were collected in the FAA fixative solution, aspirated in the laboratory and stored at 4 °C. The stems for transcriptome and IAA content determination were frozen in liquid nitrogen and stored at −80 °C until the extraction of total RNA. The length and diameter of new branches of *Ilex verticillata* ‘Oosterwijk’ and ‘Red sprite’ were measured and counted at least three biological replicates.

### 4.2. Histological Analysis

The FAA-fixed samples were performed with different concentrations of ethanol and xylene. Then, the samples fixed in FAA were dehydrated and embedded in paraffin. Stems were sectioned for histology using a Vibratome Series 1000 (Heath Company) to a thickness of 50 to 100 μm. Next, dyeing with saffron and fast green was carried out. Finally, they were observed and photographed with a positive fluorescence microscope after sealing the slices. The xylem thickness and vessel area were measured using the Image J software every three slices. The measurement data of xylem thickness came from three randomly selected sections and the vessel area was calculated from three randomly selected sections of a certain size (yellow area in this study).

### 4.3. RNA Isolation and Illumina Sequencing

The total RNA was extracted using Trizol reagent (thermofisher, 15596018) following the manufacturer’s procedure. The quantity and purity of total RNA were analyzed by Bioanalyzer 2100 and RNA 6000 Nano LabChip Kit (Agilent, Santa Clara, CA, USA, 5067-1511) with RIN number > 7.0. Then, mRNA was purified from total RNA (5 ug) using Dynabeads Oligo(dT) (Thermo Fisher, Waltham, MA, USA) with two rounds of purification. After purification, the mRNA was fragmented into small pieces using divalent cations under an elevated temperature. Then, the average insert size for the final cDNA library was 300 ± 50 bp. Then, the 2 × 150 bp paired-end sequencing (PE150) was performed on an Illumina Novaseq™ 6000 (LC-Bio Technology CO., Ltd., Hangzhou, China) in accordance with the recommended protocol.

### 4.4. De Novo Assembly and Functional

Firstly, cutadapt software and in-house perl scripts were used to remove the reads that contained undetermined, adaptor contamination and low-quality bases. Then, the sequence quality was verified using FastQC (http://www.bioinformatics.babraham.ac.uk/projects/fastqc/, 0.11.9, accessed on 16 February 2022) including the Q20, Q30 and GC-content of the clean data. All downstream analyses were based on high quality clean data. After that, a total of G bp of cleaned, paired-end reads were produced. Then, we aligned reads of all samples to the *Ilex verticillata* reference genome using the HISAT2 (https://daehwankimlab.github.io/hisat2/, version: hisat2-2.2.1, accessed on 16 February 2022) package which initially removed a portion of the reads based on quality information accompanying each read and then mapped the reads to the reference genome.

The mapped reads were assembled using StringTie (http://ccb.jhu.edu/software/stringtie/, version: stringtie-2.1.6, accessed on 16 February 2022) with default parameters. Then, all transcriptomes of all samples were merged to reconstruct a comprehensive transcriptome using gffcompare software (http://ccb.jhu.edu/software/stringtie/gffcompare.shtml, version: gffcompare-0.9.8, accessed on 16 February 2022). After the final transcriptome was generated, StringTie and ballgown (http://www.bioconductor.org/packages/release/bioc/html/ballgown.html, accessed on 16 February 2022) were used to estimate the expression levels of all transcripts and perform expression abundance for mRNAs by calculating FPKM (fragment per kilobase of transcript per million mapped reads) value [48,49,50]. Then, all DEGs were mapped to GO (Gene Ontology) databases (http://www.geneontology.org/, accessed on 16 February 2022) and KEGG (Kyoto Encyclopedia of Genes and Genomes) databases (https://www.genome.jp/kegg/, accessed on 16 February 2022).

### 4.5. Identification of Differentially Expressed Genes

The differential expression genes (DEGs) between the sample categories were identified using the edgeR package (https://bioconductor.org/packages/release/bioc/html/edgeR.html, accessed on 16 February 2022). Usually, it is evaluated from two aspects of the difference multiple and the significant level and the differentially expressed genes are screened. Here, we use the difference multiple FC ≥ 2 or FC ≤ 0.5 (|log2 fc| ≥ 1) and *p*-value < 0.05 as the standard; the genes screened out are considered as differentially expressed genes.

### 4.6. Weighted Gene Co-Expression Network Analysis

The co-expression gene network was constructed using the WGCNA package of software in reference to the tutorial on the WGCNA official website. At first, genes that were not expressed or were poorly expressed in all samples were filtered out. Then, it was determined as to whether there were outliers according to the sample clustering tree diagram. The outliers would be filtered out. According to the filtered data for computing, module information was mined. The correlation between modules, the correlation between modules and samples and the correlation between genes and modules were calculated and then visualized. To further study the gene modules associated with stem differences, the correlation coefficients between module eigengenes and the samples were calculated. Modules with the largest correlation coefficients were selected as target gene modules in the O_P3 vs. R_P3 group. Then, GO analysis and KEGG analysis of DEGs were performed using GO databases and KEGG databases.

### 4.7. IAA Content Determination

The quantification of endogenous IAA was performed according to the method reported previously with some modification [51]. The ground powder of fresh plant tissues (about 200 mg) was extracted for overnight treatment in methanol containing [^2^H_2_]-IAA as internal standards. The crude extracts were further purified by the Oasis MCX SPE cartridge which was first activated and equilibrated with methanol, water and 0.05% formic acid (FA). After the samples loading into the cartridge, the cartridge was sequentially washed with 5% FA and water. Finally, IAA was eluted with methanol and analyzed on a liquid chromatography-tandem mass spectrometry system comprising an ACQUITY UPLC (Waters) and Qtrap 6500 system (AB SCIEX) equipped with an electrospray ionization (ESI) source. The separation of IAA was achieved on a Waters ACQUITY UPLC BEH C18 column (100 × 2.1 mm i.d., 1.7 µm) with mobile solvents of 0.1% FA in water (solvent A) and acetonitrile (solvent B) at a programmed gradient from 95:5 (*v*/*v*) A:B to 5:95 (*v*/*v*) A:B within 5 min. The multiple reaction monitoring (MRM) transitions for IAA and [^2^H_2_]-IAA were as follows: IAA 176.2 > 130.1 and [^2^H_2_]-IAA 178.2 > 132.0. The analysis was performed on three biological replicates.

## 5. Conclusions

In this study, stem morphological observations and comparative transcriptome analysis were performed on stem tissues of ‘Oosterwijk’ and ‘Red sprite’ at three reproductive stages to explore the key genes responsible for the differences in stem growth between these two varieties. The O_P1 vs. R_P1, O_P2 vs. R_P2 and O_P3 vs. R_P3 groups contain 3908, 3630 and 2703 DEGs, respectively. The enriched metabolic pathways included phenylpropanoid biosynthesis, phenylalanine metabolism, tryptophan metabolism, plant hormone signal pathway and phenylalanine, tyrosine and tryptophan biosynthesis. The results of endogenous auxin determination showed that the auxin content in the stem tissue of ‘Oosterwijk’ was higher and the DEGs related to auxin were also identified as candidate genes for stem growth difference. These further illustrated that the change of auxin content and the expression difference of auxin-related genes may be the key factors for the difference between the two varieties of stems. This study can serve as a basis for the identification and functional study of auxin and the stem development of *Ilex verticillata* and help to illustrate the molecular mechanism of *Ilex verticillata* stem growth difference, which is expected to breed new *Ilex verticillata* varieties.

## Data Availability

Not applicable.

## Supplementary Materials

The following supporting information can be downloaded at: https://www.mdpi.com/article/10.3390/plants12101941/s1. Figure S1: Phenotypic observation of the autumn new shoots of ‘Oosterwijk’ and ‘Red sprite’. Figure S2: Cytological section observation of new branches in current year of *Ilex verticillata* ‘Oosterwijk’ and ‘Red sprite’. Figure S3: DEGs volcano plot in each comparison group. Figure S4: GO functional enrichment analysis of DEGs in O_P2 vs. O_P1, O_P3 vs. O_P2 and O_P3 vs. O_P1 groups. Figure S5: GO functional enrichment analysis of DEGs in R_P2 vs. R_P1, R_P3 vs. R_P2 and R_P3 vs. R_P1 groups. Figure S6: GO functional enrichment analysis of DEGs in O_P1 vs. R_P1, O_P2 vs. R_P2 and O_P3 vs. R_P3 groups. Figure S7: KEGG functional enrichment analysis of DEGs in O_P2 vs. O_P1, O_P3 vs. O_P2 and O_P3 vs. O_P1 groups. Figure S8: KEGG functional enrichment analysis of DEGs in R_P2 vs. R_P1, R_P3 vs. R_P2 and R_P3 vs. R_P1 groups. Figure S9: KEGG functional enrichment analysis of DEGs in O_P1 vs. R_P1, O_P2 vs. R_P2 and O_P3 vs. R_P3 groups. Table S1: Sample sequencing data collation and align information between samples and *Ilex verticillata.* Table S2: Number of genes in 25 modules.
