# Supplementary material for: Comparative Microscopic, Transcriptome and IAA Content Analyses Reveal the Stem Growth Variations in Two Cultivars Ilex verticillata
Source: Plants (Basel). 2023 May 10;12(10):1941. doi: 10.3390/plants12101941 (PMC10220661; doi:10.3390/plants12101941)
Supplement: Supplementary file 1 [file plants-12-01941-s001.zip › plants-2365620-supplementary.pdf]

Supplementary Materials

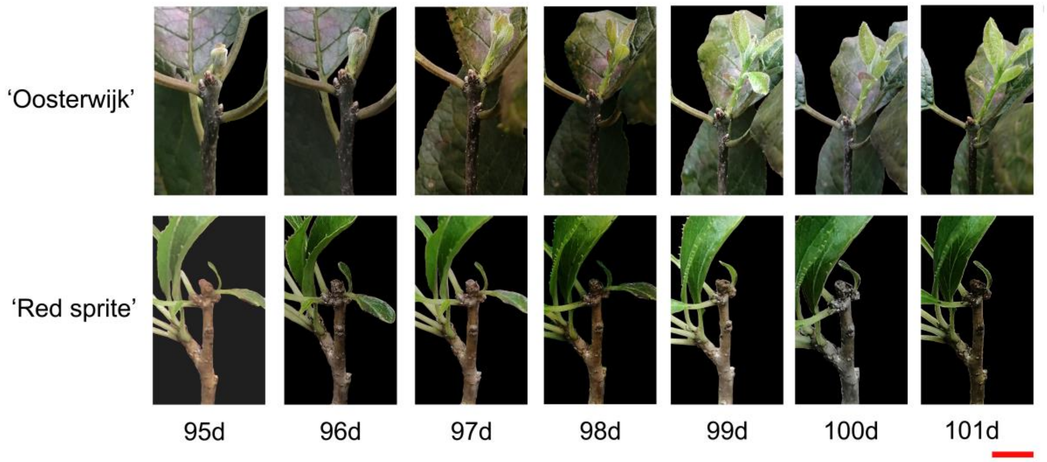

Supplementary Figure S1. Phenotypic observation of the autumn new shoots of 'Oosterwijk' and 'Red sprite'. Phenotypic differences in new shoots between 'Oosterwijk' and 'Red sprite' were observed from the 95d to 101d (29th May 2021 is used as the starting point for time calculations). Scale bar=1cm.

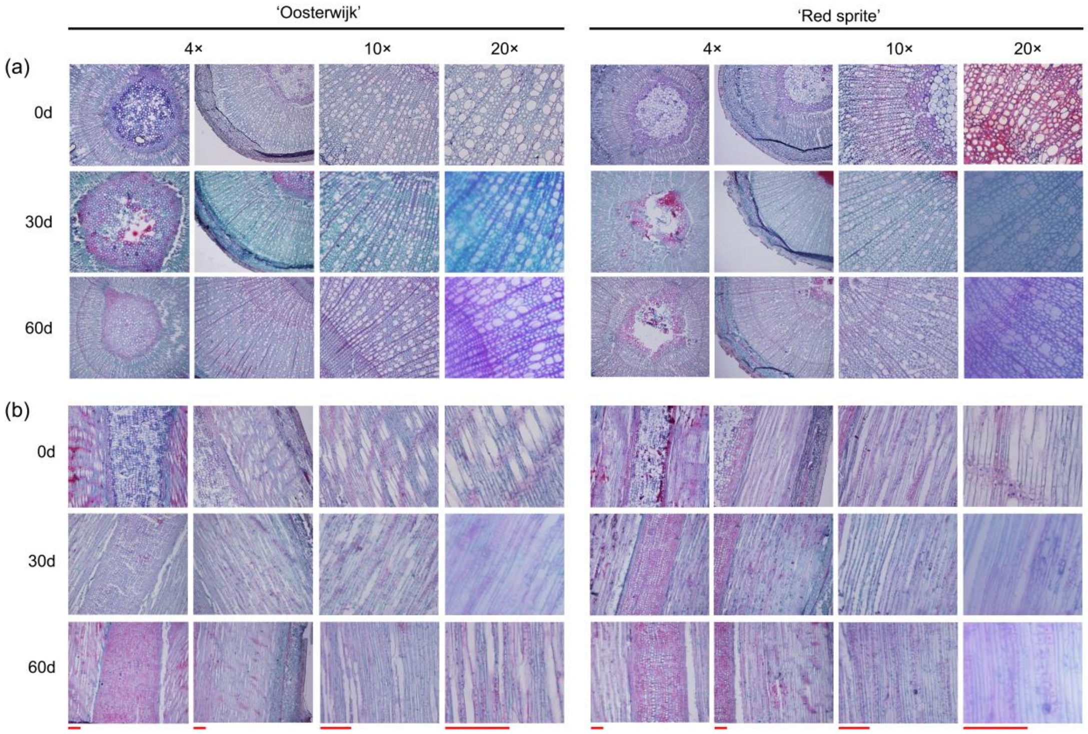

Supplementary Figure S2. Cytological section observation of new branches in current year of *Ilex verticillata* 'Oosterwijk' and 'Red sprite'. Scale bar=200μm. (a) the cross sections of the stems. The sections were observed under positive fluorescence microscope 4×, 10× and 20× magnification, respectively. Scale bar=200μm. (b) the longitudinal sections of the stems. The sections were observed under positive fluorescence microscope at 4×, 10× and 20× magnification, respectively. Scale bar=200μm.

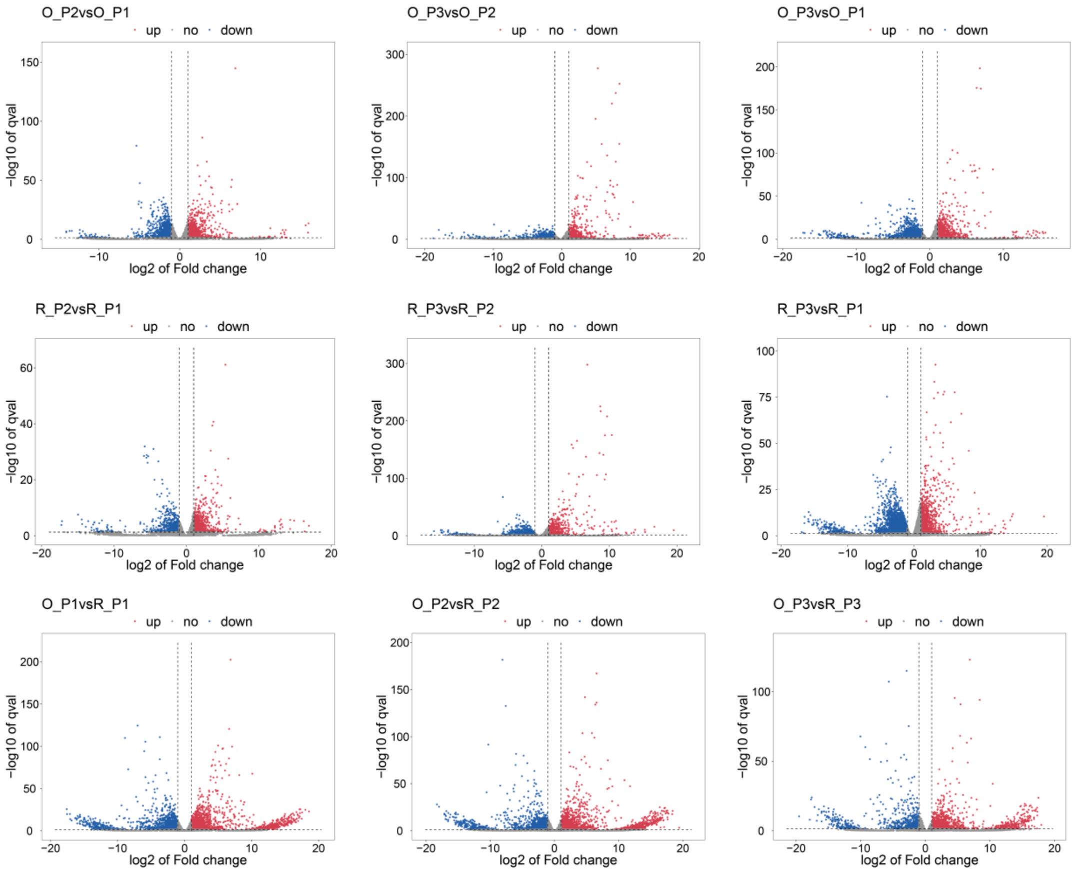

Supplementary Figure S3. DEGs volcano plot in each comparison group. O_P2vsO_P1, O_P3vsO_P2, O_P3vsO_P1, R_P2vsR_P1, R_P3vsR_P2, R_P3vsR_P1, O_P1vsR_P1, O_P2vsR_P2 and O_P3vsR_P3. O indicates 'Oosterwijk', R indicates 'Red sprite'; P1, P2 and P3 indicate Phase1 (0d), Phase2 (30d) and Phase3 (60d). The up-regulated and down- regulated genes are represented by red and blue dots, respectively, and the gray dots represent genes with no significant difference in expression.

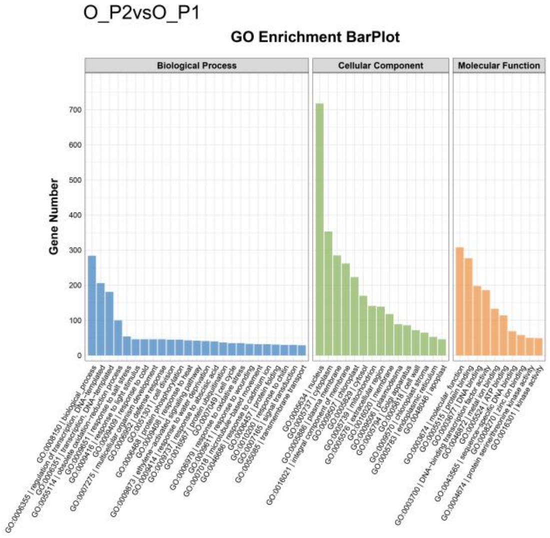

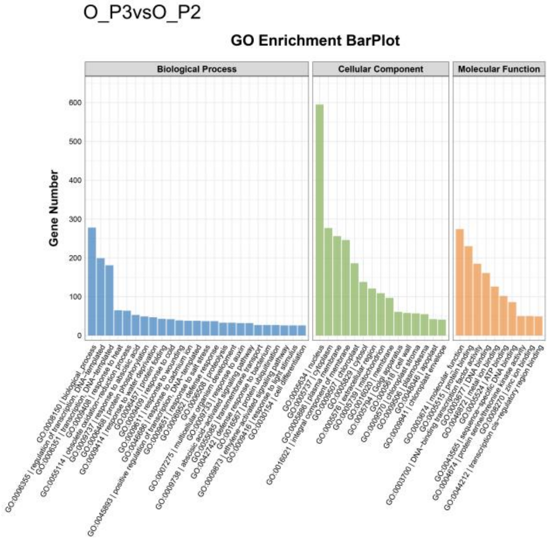

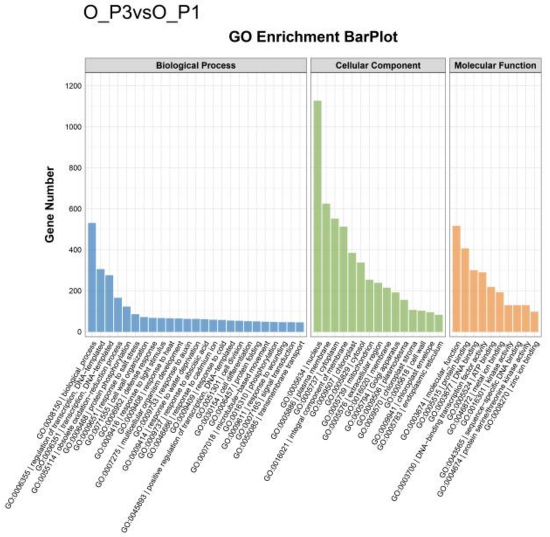

Supplementary Figure S4. GO functional enrichment analysis of DEGs in O_P2vsO_P1, O_P3vsO_P2 and O_P3vsO_P1 groups. O indicates 'Oosterwijk'; P1, P2 and P3 indicate Phase1 (0d), Phase2 (30d) and Phase3 (60d).

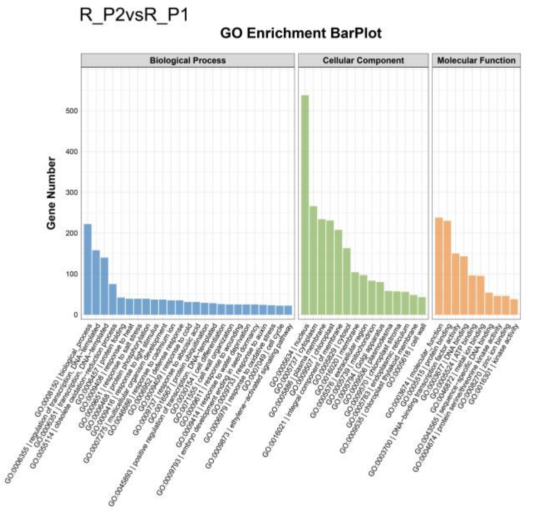

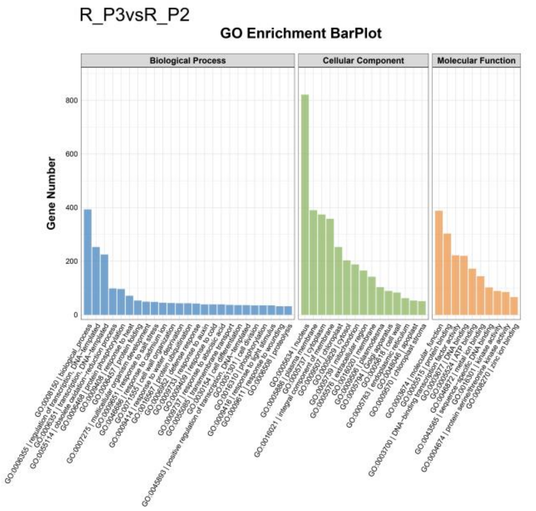

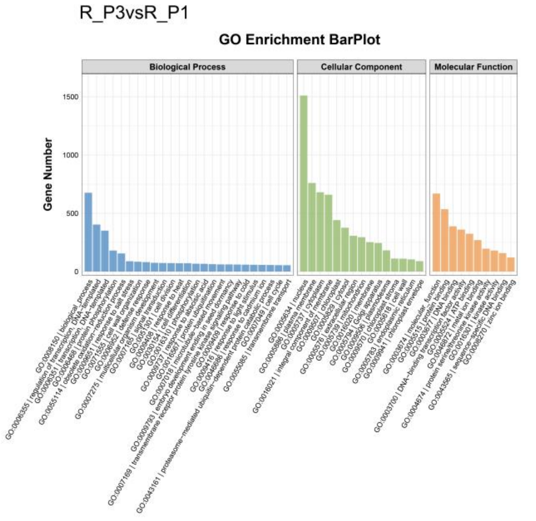

Supplementary Figure S5. GO functional enrichment analysis of DEGs in R_P2vsR_P1, R_P3vsR_P2 and R_P3vsR_P1 groups. R indicates 'Red sprite'; P1, P2 and P3 indicate Phase1 (0d), Phase2 (30d) and Phase3 (60d).

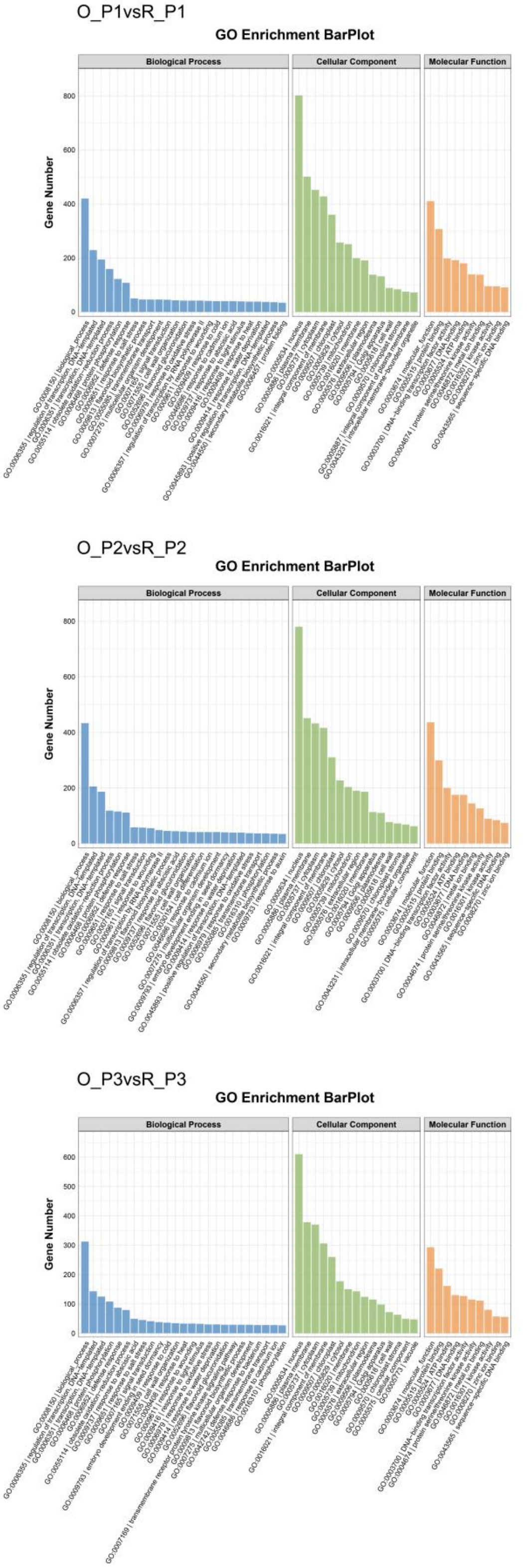

Supplementary Figure S6. GO functional enrichment analysis of DEGs in O_P1vsR_P1, O_P2vsR_P2 and O_P3vsR_P3 groups. O indicates 'Oosterwijk', R indicates 'Red sprite'. P1, P2 and P3 indicate Phase1 (0d), Phase2 (30d) and Phase3 (60d).

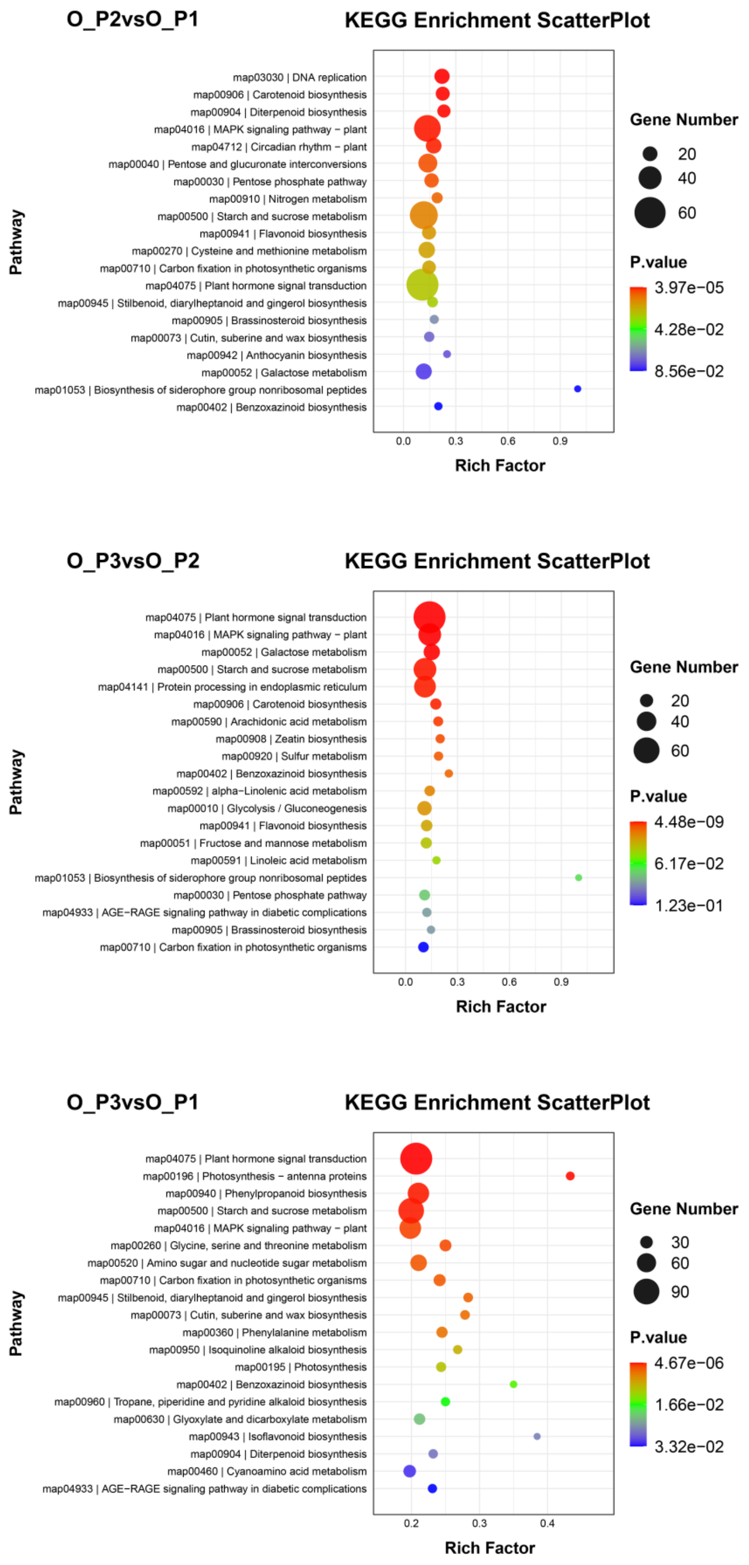

Supplementary Figure S7. KEGG functional enrichment analysis of DEGs in O_P2vsO_P1, O_P3vsO_P2 and O_P3vsO_P1 groups. O indicates 'Oosterwijk'; P1, P2 and P3 indicate Phase1 (0d), Phase2 (30d) and Phase3 (60d).

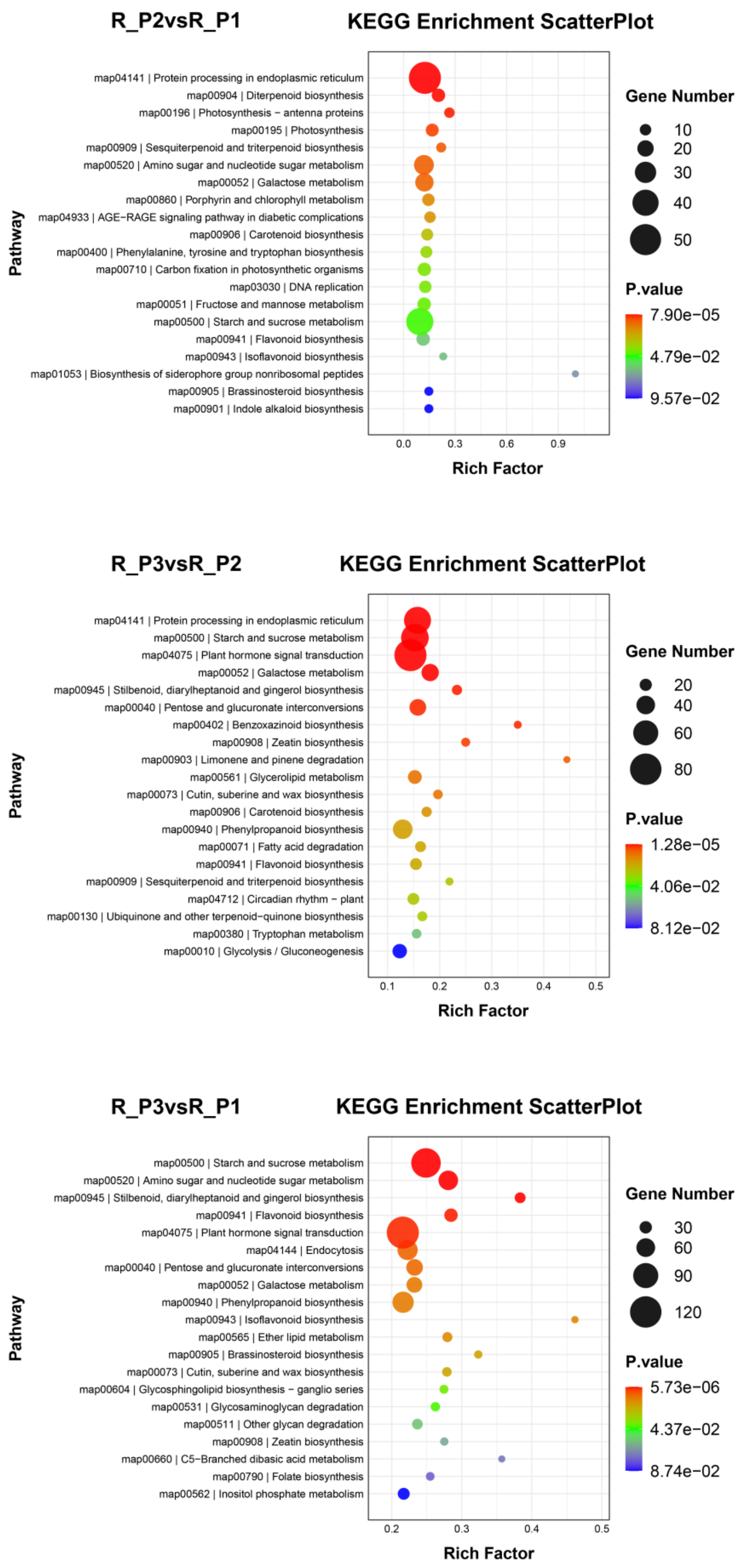

Supplementary Figure S8. KEGG functional enrichment analysis of DEGs in R_P2vsR_P1, R_P3vsR_P2 and R_P3vsR_P1 groups. R indicates 'Red sprite'; P1, P2 and P3 indicate Phase1 (0d), Phase2 (30d) and Phase3 (60d).

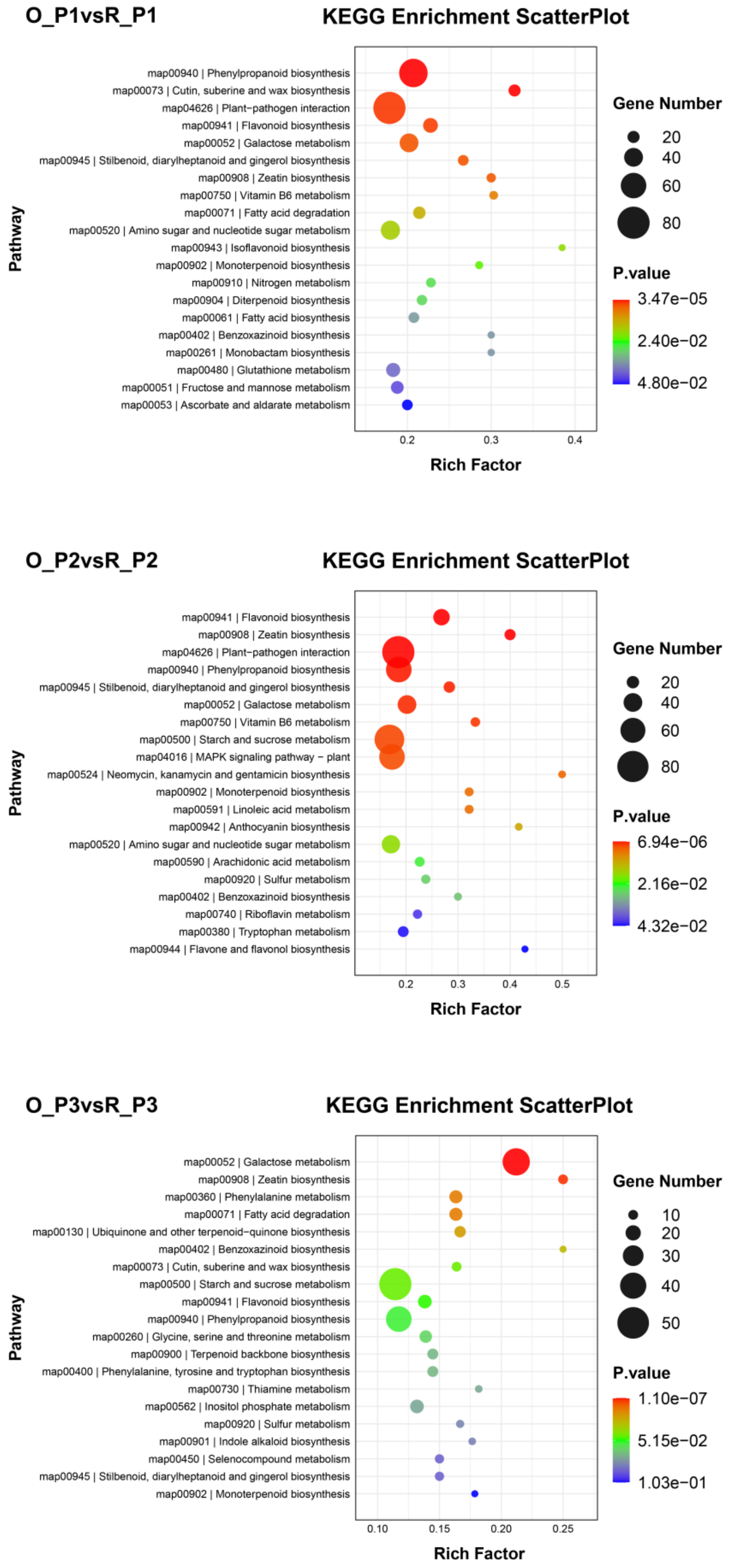

Supplementary Figure S9. KEGG functional enrichment analysis of DEGs in O_P1vsR_P1, O_P2vsR_P2 and O_P3vsR_P3 groups. O indicates 'Oosterwijk', R indicates 'Red sprite'; P1, P2 and P3 indicate Phase1 (0d), Phase2 (30d) and Phase3 (60d).

Supplementary Table S1. Sample sequencing data collation and align information between samples and *Ilex verticillata.*

Sample	Valid Reads	Valid Ratio (%)	Valid data base	Mapped Reads rate (%)
O_P1_1	43051878	94.76	6.46G	87.87
O_P1_2	45824950	95.16	6.87G	88.03
O_P1_3	47357354	92.24	7.10G	88.57
O_P2_1	44570834	94.29	6.69G	87.65
O_P2_2	42866468	95.13	6.43G	87.63
O_P2_3	45874352	95.18	6.88G	87.49
O_P3_1	44424442	94.26	6.66G	87.02
O_P3_2	42828750	95.23	6.42G	85.71
O_P3_3	45800894	95.15	6.87G	86.26
R_P1_1	45216334	95.57	6.78G	84.05
R_P1_2	39501936	92.43	5.93G	83.95
R_P1_3	44542428	95.13	6.68G	84.49
R_P2_1	39482178	92.98	5.92G	82.49
R_P2_2	42653880	91.49	6.40G	83.28
R_P2_3	45285478	94.93	6.79G	83.31
R_P3_1	38128536	93.12	5.72G	81.06
R_P3_2	31673784	93.96	4.75G	82.57
R_P3_3	40175710	94.40	6.03G	83.19

[S1] O_P_1, O_P_2, O_P_3 represent the stems of 'Oosterwijk' at 0d, 30d and 60d, respectively; R_P_1, R_P_2, R_P_3 represent the stems of 'Red sprite' at 0d, 30d and 60d, respectively. The last number for each cell represents the three biological replicates

Supplementary Table S2. Numbers of genes in 25 modules.

Module color	Gene numbers
Black	1170
Blue	4131
Brown	2570
Cyan	413
Darkgreen	79
Darkgrey	37
Darkred	88
Darkturquoise	53
Green	1411
Greenyellow	638
Grey	24
Grey60	233
Lightcyan	246
Lightgreen	212
Lightyellow	181
Magenta	801
Midnightblue	329
Pink	1158
Purple	680
Red	1302
Royalblue	89
Salmon	461
Tan	492
Turquoise	5959
Yellow	1438
